# Identification of Cysteine Proteases and Screening of Cysteine Protease Inhibitors in Biological Samples by a Two-Dimensional Gel System of Zymography and Reverse Zymography

**Published:** 2007-11-18

**Authors:** Eiichi Saitoh, Shinya Yamamoto, Eishiro Okamoto, Yoshimi Hayakawa, Takashi Hoshino, Ritsuko Sato, Satoko Isemura, Sadami Ohtsubo, Masayuki Taniguchi

**Affiliations:** 1 Graduate School of Technology, Niigata Institute of Technology, Kashiwazaki, Niigata 945-1195, Japan.; 2 The Nippon Dental University College at Niigata, Niigata, Niigata 951-8580, Japan.; 3 Food Research Center, Niigata Agricultural Research Institute, Kamo, Niigata 959-1381.; 4 Department of Material Science and Technology, Faculty of Engineering, Niigata University, Niigata, Niigata 951-2181, Japan.

**Keywords:** Broad bean lectin, Cysteine proteases, Cysteine protease inhibitors, Reverse zymography, Two-dimensional gel electrophoresis, Zymography

## Abstract

We have developed a two-dimensional (2D-) gel system of zymography and reverse zymography for the detection and characterization of proteases and protease inhibitors. Isoelectric focusing (IEF) agarose gels with pH gradients were employed for separation in the first-dimension and sodium dodecyl sulfate (SDS)-polyacrylamide gel copolymerized with gelatin used for the second dimension. Proteases and protease inhibitors separated by IEF gel were applied on the second gel without trichloroacetic acid (TCA) fixation. Protease activity in the 2D-gel was visualized as transparent spots where gelatin substrate was digested after commassie brilliant blue (CBB) staining. Some of the transparent spots from the skin mucus extract of rainbow trout were determined to be a cysteine protease through use of E-64 or CA-074. In the reverse zymography technique, the gel was incubated with papain solution at 37 ºC for 18 h. Cysteine protease inhibitors from broad bean seeds were detected as clear blue spots after CBB staining. The amino (N-) terminal sequences of four papain inhibitor spots thus detected were demonstrated to be identical to that of favin β chain, a broad bean lectin. Taken together, our system can be considered to be an efficient technique for discovering and characterizing new proteases and protease inhibitors in biological samples. This is the first report describing a 2D-gel system of zymography and reverse zymography.

## Introduction

Endogenous proteases and protease inhibitors play an essential role in maintaining life in all organisms. Protease inhibitors are proposed to regulate the proteolytic activity in a wide variety of physiological and pathological processes *in vivo* (Monard, 1988; Solomon et al. 1999;Turk et al. 2002; Katunuma et al. 2003).

Zymography and reverse zymography of one-dimensional (1D-) gel systems have been extensively employed as a convenient technique for the identification and characterization of proteases and protease inhibitors (Heussen et al. 1980; Yasothornsrikul and Hook, 2000; Ohashi et al. 2003; Le and Katunuma 2004; Katunuma et al. 2005; Le et al. 2005; Saitoh et al. 2005). These methods are based on the separation of proteases and protease inhibitors in biological samples on an SDS-polyacrylamide gel (Laemmli, 1970) containing substrates such as gelatin, casein or fluorescent compounds copolymerized in gel. For zymography, the substrate in gel is digested at 37 °C for an optimal incubation period by the proteases separated. The protease activity located where the substrate is digested, can be visualized as a transparent band on a blue background after protein staining. For reverse zymography, the substrate in gel is digested by the target protease solution at 37 °C for an optimal incubation period. Undigested substrate remains, where a protease inhibitor molecule is located and can be stained as a blue band.

Numerous contributions by the technique have been made in the field of biological and medical sciences, however, the 1D-gel system has several limitations. It cannot provide high resolution of proteins from samples nor information on the isoelectric point of separated proteins. Moreover, mutation cannot be adequately analyzed by means of any 1D-gel system. Included in progress in the development of tools for proteomic analysis is the commercial availability of a variety of useful IEF gels with immobilized pH gradients for 2D-gel electrophoresis (O’Farrell, 1975; Klose, 1975; Taylor and Coorssen, 2006). This progress has permitted us to develop a new, useful system of zymography (or reverse zymography) combined with 2D-gel electrophoresis for the identification and characterization of proteases (or protease inhibitors).

In this paper, we demonstrate the use of this system in the characterization of cysteine proteases from the skin mucus extract of rainbow trout (*Oncrhynchus mykiss*) and screening of cysteine protease inhibitors from an extract of broad bean (*Vicia faba*) seeds.

## Experimental Procedures

### Materials

Papain (2 × crystallized) [EC 3.4.22.2] from papaya latex (28 mg protein/ml, 27 U/mg) was obtained from Sigma Chemical (St. Louis, MO, U.S.A.). Benzyloxycarbonyl (Z)-Phe-Arg-methylcoumaryl-7-amide (MCA), (L-trans-Carboxyoxirane-2-carbonyl)-L- leucylagmatine (E-64), and (L-trans-(Propylcarbamoyl)-oxirane-2-carbonyl)-L-isoleucyl-L-proline (CA-074) were from Peptide Institute Inc, Osaka. Molecular weight markers were from Bio-Rad Chemical Co (Richmond, CA, U.S.A.). Immobilon^™^ polyvinylidendifluoride (PVDF) filter was from Millipore Co (Bedford, MA, U.S.A.). IEF disc agarose gels with immobilized pH gradients were purchased from ATTO Corp (Tokyo, Japan). All other reagents used were of analytical grade.

### Preparation of the skin mucus extract of rainbow trout

Living rainbow trout (body weight, 600–700g; total length, 50–60 cm) were obtained from Niigata prefectural inland water fishery experimental station and were immediately frozen in dry-ice. The skin mucus was collected by scraping the body surface layers of eight rainbow trout with a spatula, resuspended in 600 ml of 0.01 M sodium phosphate buffer (pH 7.0) containing 150 mM NaCl and homogenized on ice with a homogenizer (Model PT-1200E, KINEMATICA, Lucerne, Switzerland). The precipitate in the suspension was removed by centrifugation at 12,000 × g (4 °C) for 30 min (Model SRX-201, TOMY Corp, Tokyo, Japan). The supernatant was collected and stored as the starting material at − 20 °C until use.

### Preparation of cysteine protease inhibitor fractions of broad bean seeds

Broad beans were purchased from commercial suppliers. The seeds (195g) were shelled and ground into a paste with a pestle and mortar. The paste was further homogenized on ice with 100 ml of 0.01 M sodium phosphate buffer (pH 7.0) containing 150 mM NaCl. The homogenate was then centrifuged at 12, 000 × g (4 °C) for 30 min and the supernatant fraction was stored as a starting material at −20 °C until use. The fractions of cysteine protease inhibitor were obtained from the starting material by gel filtration on a Sephacryl S-200 column (1.2 × 110 cm) equilibrated with 0.01 M ammonium bicarbonate at 4 °C.

### Measuring of the activities for cysteine protease and cysteine protease inhibitor

The active concentration of papain was determined by titration with E-64 using Z-Phe-Arg-MCA as a substrate according to the method of Barrett and Kirschke (1981). Proteolytic activities of the skin mucus extract of rainbow trout, the proteolytic inhibitory activities of E-64 (or CA-074) and the papain-inhibitory activity of the fractions from the broad bean seed extract eluted by column chromatography were assayed using a Hitachi fluorescence spectrophotometer F-2500 with Z-Phe-Arg-MCA as a substrate (Barrett and Kirschke, 1981). Assays were performed in a total volume of 2 ml. The buffers used were: (i) 0.1 M citric acid-NaOH (pH 3.5–5.5); (ii) 0.1 M NaH_2_PO_4_-NaOH (pH 6.0–7.5); (iii) 0.1 M Tris-HCl (pH 8.0–9.0); and (iv) 0.1 M Gly-NaOH (pH 9.5). All the buffers included 0.05 % Brij 35 (final concentration) and 1 mM EDTA (final conc). 2 mM DTT (final conc) was sometimes added to the buffers as indicated in the main text. The amount of MCA librated from the substrate was determined using excitation and emission wavelengths of 380 nm and 460 nm, respectively. The concentration of protein in biological samples was determined by the method of Bradford (1976) using a Bio-Rad assay kit with bovine serum albumin as the standard.

### 2D-gel electrophoresis for zymography and reverse zymography

The biological samples were separated using a centrifuge (Model Himac-CS-100GX, Hitachi Corp, Ibaraki, Japan) at 110,000 × g (4 °C) for 30 min and used in IEF separation on agarose disc gels (total length, 7.5 cm; diameter, 2.5 mm) having immobilized pH gradients of 3–8 or 3–10). IEF was performed with discRun (Model AE-6541, ATTO) at 4 °C. A sample aliquot of 20 μl (protein content of 18.8 mg/ml for the skin mucus extract of rainbow trout and 33.5 mg/ml for the broad bean seeds extract) were mixed with 20 μl of 10% glycerol and then directly applied to the agarose disc gels. For the investigation of the effect on proteolytic activities of synthetic inhibitors, the skin mucus extract of rainbow trout (20 μl) was incubated with an excess amount of E-64 (final conc, 440 μM) or of CA-074 (final conc, 440 μM) for 5 min at room temperature before loading on the IEF gels. After the IEF was completed, the agarose gel was immediately placed on a 12 % SDS-polyacrylamide slab gel (9 × 8 × 0.1 cm) copolymerized with or without 0.1 % gelatin (Bio-Rad). The IEF gel was sometimes fixed with 10% TCA for 3 min before placing on the 2D-gel. SDS-polyacrylamide gel electrophoresis (PAGE) was then performed at 4 °C according to the method of Laemmli (1970) (Model AE-6500, ATTO).

After SDS-PAGE was completed, the slab gel was removed and shaken at room temperature for 15 min in 2.5% Triton X-100 (twice) to remove SDS. The gel was then rinsed with distilled water twice and incubated at 37 °C in 15 ml of the zymography-developing buffer for 24 h. The zymography-developing buffer for cysteine proteases with an acidic optimal pH consisted of 0.1 M sodium citrate (pH 4.0), 0.05% Brij 35, 1 mM EDTA, and 2 mM DTT. For cysteine proteases with a neutral optimal pH, 0.1 M sodium phosphate buffer (pH 6.8) containing 0.05% Brij 35, 1 mM EDTA and 2 mM DTT was used as the developing buffer.

To 15 ml of the zymography-developing buffer, 7.56–15.1 mU of papain (80% active) was added to make the reverse-zymography-developing buffer and the gel was incubated at 37 °C in this buffer for 18 h.

**Figure 1.**
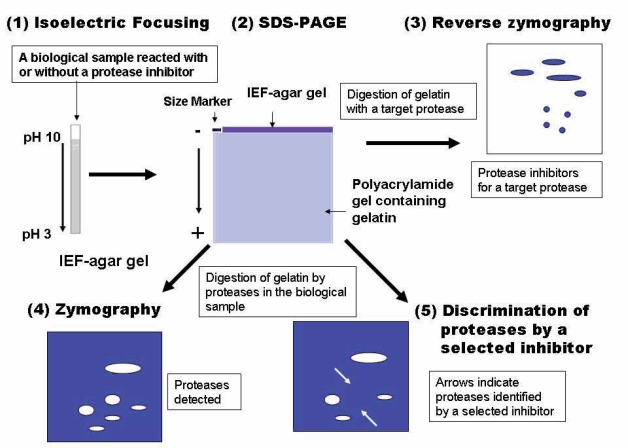
Schematic illustration of the 2D-gel zymography and reverse zymography system.

After the zymography (or reverse zymography) developing procedure was finished, the gel was stained with CBB-R350 and destained with a solution (50% distilled water, 40% methanol, and 10% acetic acid). Occasionally, the gel was fixed with 10% TCA before CBB-R350 staining. Figure 1 summarizes the procedure and principle of the 2D-gel system of zymography and reverse zymography.

### Determination of N-terminal amino acid sequences and homology analysis

The cysteine protease inhibitor spots to be sequenced were transferred to a PVDF filter using a trans-blot apparatus (ATTO, Model AE-6677). The N-terminal sequence of proteins was determined by automated Edman degradation with an Applied Biosystems model 492 sequencer. Homology analysis of the determined sequence was performed by searching the BLAST database (http://www.ddbj.nig.ac.jp/Welcome-j.html).

## Results and Discussion

### Examination of proteolytic activities in the skin mucus extract of rainbow trout

In the present study, we focused on the identification and characterization of cysteine proteases and cysteine protease inhibitors since we are interested in the biological regulation of cysteine proteases by cystatins (Isemura et al. 1984, 1991; Saitoh and Isemura, 1994; Saitoh et al. 1998, 2005; Ohtsubo et al. 2005; Akiba et al. 2006).

This study describes the investigation of cysteine proteases in the epidermis of rainbow trout using a 2D-gel system of zymography, since cysteine proteases (cathepsins B or L) on the surface of epidermal cell layers are proposed to play important roles in the defense mechanisms of fishes such as the Japanese eel (Aranishi, 1997, 1999) and Atlantic salmon (Tahtinen et al. 2002).

**Figure 2.**
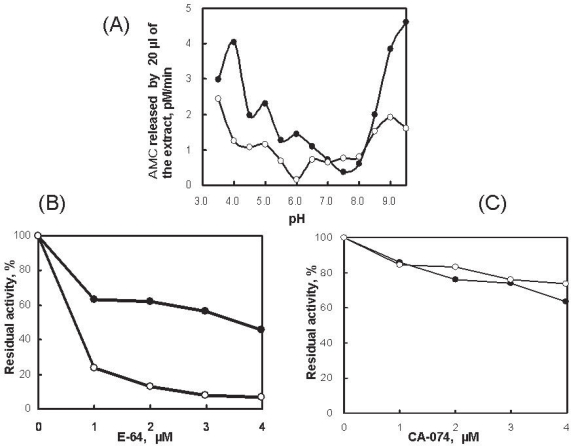
Characterization of Z-Phe-Arg-MCA hydrolyzing-activity in skin mucus extract of rainbow trout. **A**) Z-Phe-Arg-MCA hydrolyzing-activity at different pHs in the presence (closed circles) and absence (open circles) of 2 mM DTT. **B**) Inhibition of Z-Phe-Arg-MCA hydrolyzing-activity by E-64 at pH 6.8 (closed circles) and pH 4.0 (open circles) in the presence of 2 mM DTT. **C**) Inhibition of Z-Phe-Arg-MCA hydrolyzing-activity by CA-074 at pH 6.8 in the presence of 4 mM DTT (closed circles) and pH 4.0 (open circles).

The skin mucus extract of rainbow trout shows hydrolyzing-activity towards Z-Phe-Arg-MCA under reducing conditions at pH 3.5–9.5, as shown in Figure 2(A). This result is evidence for the presence of cysteine proteases such as cathepsins. The fluorescent substrate hydrolyzing-activity was also detected under non-reducing conditions, suggesting the presence of proteases other than cysteine proteases in the skin mucus extract. Figs. 2(B) and 2(C) show the inhibition of proteolytic activity in the extract by E-64 (a specific inhibitor for cysteine proteases) and CA-074 (a specific inhibitor for cathepsin B) at pH 4.0 and pH 6.8. These inhibition curves further demonstrate the presence of a cysteine protease in the extract. The proteases in the skin extract were then analyzed using a 2D-gel zymography system.

### Detection and characterization of proteolytic activities in the skin mucus extract of rainbow trout using a 2D-gel zymography system

Initially, great attention was paid to the preparation of the biological samples in order to retain the proteolytic activities during the protein separation on the 2D-gel zymography system. For general 2D-PAGE, several sample solubilization buffer systems have been proposed (Herbert, 1999; Natarajan et al. 2005) for improving protein resolution. However, the chemicals in the solubilization buffers such as thiourea, urea, TCA and α-cyanohydroxycinnamic acid are likely to destroy the activity of proteases and protease inhibitors in the samples. Therefore, we have tried to introduce a simple buffer system for the extraction of proteins as described in the Materials and Methods section. To avoid the inactivation of proteolytic activity, care was also taken with the fixation procedure of the agarose gel after IEF.

**Figure 3.**
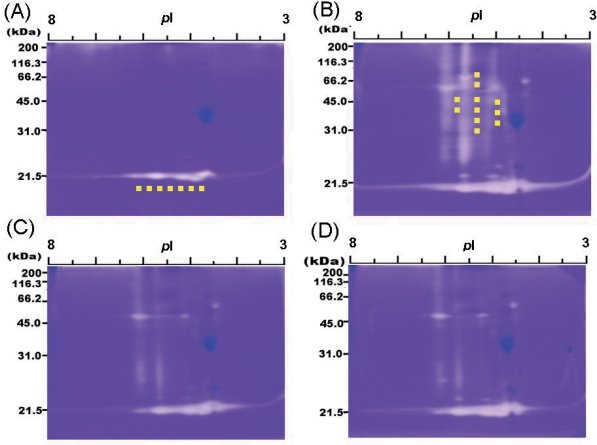
Detection and basic characterization of proteases in skin mucus extract of rainbow trout at pH 6.8 by 2D-gel zymography. **A**) Detection of protease activities with TCA fixation after IEF. **B**) Without TCA fixation after IEF. **C**) Identification of cysteine proteases by treatment with E-64 without TCA fixation after IEF. **D**) Identification of cysteine proteases by treatment with CA-074 without TCA fixation after IEF.

**Figure 4.**
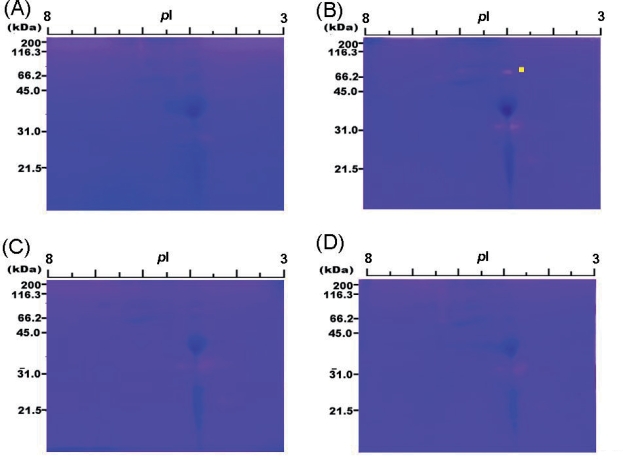
Detection and identification of proteases in the skin mucus extract of rainbow trout at pH 4.0 by 2D-gel zymography. **A**) Detection of protease activities with TCA fixation after IEF. **B**) Without TCA fixation after IEF. **C**) Identification of cysteine proteases by treatment with E-64 without TCA fixation after IEF. **D**) Identification of cysteine proteases by treatment with CA-074 without TCA fixation after IEF.

Figures 3 and 4 show the detection and characterization of proteolytic activities in the skin mucus extracts at pH 6.8 and pH 4.0 respectively. Numerous proteolytic activities were visualized as transparent spots on the blue background at pH 6.8 (Fig. 3B), whilst only a limited spot was observed at pH 4.0 (Fig. 4B). These results are inconsistent with the MCA-peptide hydrolyzing activities demonstrated in Figure 2. This conflict may be due to differences in affinity between proteases in the extract and substrates used.

Comparison of Figures 3B and 4B with Figures 3A and 4A, demonstrates that two types of spot can be distinguished from each other by the fixation of agarose gels with TCA. Using this fixation technique one type of spot disappears while the other remains. The multiple spots (highlighted by dots) around molecular masses at 21.5 kDa (pI, 4.8–6.3) are found to retain considerable activity and resist the TCA treatment (see Fig. 3A). Some of these spots did not disappear even when treated with E-64 (Fig. 3C) or CA-074 (Fig. 3D), thus defining the spots as proteases other than cysteine proteases. These spots were not observed at pH 4.0 (Fig. 4A).

A variety of spots (marked with dots) disappeared through inhibition with E-64 and CA-074, indicating that the present system is a useful method for identification of cysteine proteases in biological samples, as shown in Figures 3C, 3D, 4C, and 4D. It seems that this system is also applicable to initial characterization of proteolytic activities other than cysteine proteases if selective inhibitors of serine proteases, aspartic proteases and matrix proteases are chosen.

### Characterization of cysteine protease inhibitors in extract of broad bean seeds

Serine protease inhibitors in legume seeds have been extensively studied and well characterized (Birk, 2003). However, the information on cysteine protease inhibitors in legume seeds is limited at present to soyacystatin, cystatins (L1, R1, and N2) and the low molecular mass protein inhibitor of cysteine proteases from soybean seeds (Brzin et al. 1990; Misaka et al. 1996; Lalitha S et al. 2005). Therefore, we aimed to screen and characterize cysteine protease inhibitors from legume seeds using the 2D-gel system of reverse zymography. In the first stage of this study, we initiated a search for cysteine protease inhibitors from broad bean seeds.

**Figure 5.**
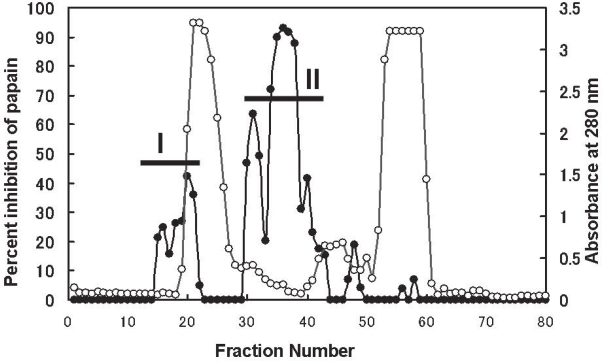
Fractionation of broad bean seed extract on a Sephacryl S-200 column. Fractions of 4.0 ml were collected. Open circles indicate the absorbance at 280 nm. Closed circles denote percent inhibition of papain.

The extract from broad bean seeds was centrifuged at 110, 000 × g (4 °C) for 30 min and then 5 ml of the supernatant (165.5 mg of protein) was applied to a Sephacryl S-200 column. Strong papain-inhibitory activities were found in fractions 15–21 and fractions 30–42 using a fluorescent substrate based assay, as shown Figure 5. The pooled eluates from both fractions were termed I and II respectively. The two fractions were confirmed as containing detectable amounts of papain inhibitors by reverse zymography on a 1D-gel system (data not shown).

### Detection and characterization of cysteine protease inhibitors from broad bean seeds using the 2D-gel reverse zymography system

Fractions I (394 μg of protein) and II (90 μg of protein) were loaded without any treatment onto IEF agarose gels with a pH range of 3–10. After IEF was completed, the gel was carefully loaded onto the second dimension 12% polyacrylamide gel immediately without TCA fixation to avoid protein denaturation.

**Figure 6.**
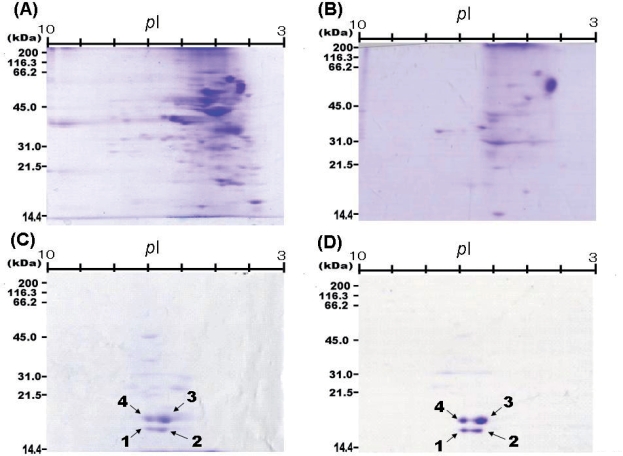
Separation and detection of proteins and cysteine protease papain inhibitors in fractions (I and II) by 2D-gel system. (**A**) Detection of proteins in fraction I; (**B**) Detection of proteins in fraction II; (**C**) Detection of papain inhibitors in fraction I by reverse zymography; (**D**) Detection of papain inhibitors in fraction II by reverse zymography.

The separation patterns of proteins obtained after 2D-gel electrophoresis of fractions I and II are shown in Figures 6A and 6C respectively. The reverse zymograms corresponding to Figures 6A and 6C are shown in Figs. 6B and 6D respectively. The papain-inhibitory spots in Figures 6B and 6D remained even when papain activity was increased from 7.56 to 15.1 mU. The multiple papain-inhibitory blue spots in fraction I were thus separated and detected (see Fig. 6B). Four papain-inhibitory spots with a higher intensity were found when fraction II was analyzed by reverse zymography, as seen in Figure 6D. The papain-inhibitory spots were then numbered 1, 2, 3, and 4. To determine the N-terminal sequences of the spots, the corresponding protein spots in Figure 6C were transferred onto a PVDF filter electrophoretically. Automated Edman degradation of the four protein spots showed all four to have an identical sequence (TDEITSFSIP-). Surprisingly, the sequence was found to be identical to the N-terminal 10 residues of favin β chain (Hopp et al. 1982; Genbank Accession No AJ 438490), suggesting the discovery of a novel cysteine protease inhibitor with lectin activity. However, the molecular masses of the four protein spots (16–18 kDa) are clearly lower than that of the full-sized form of favin β chain (26 kDa). This deviation may suggest that the four proteins could be fragments derived from the full-sized form of favin β chain.

A cysteine protease inhibitor with lectin activity (AJL2/Eel-CPI-1) has also been found in the skin mucus of the Japanese eel (Saitoh et al. 2005). Details of the cysteine protease inhibitory mechanisms by these protein spots are now under investigation. The result obtained here suggests that our system could make a contribution to this unexpected discovery in the field of protease inhibitors.

Although our protocols have clearly identified proteases and protease inhibitors in cytosolic fractions, it has been somewhat dogmatically considered unsuited to the resolution of those in membrane fractions.

## Concluding Remarks

Based on the results, we conclude that the 2D-zymography system and reverse zymography system described here is an efficient and reliable method for the identification and characterization of proteases and protease inhibitors in biological samples.
